# Supplementary material for: Development of a computer-aided design software for the quantitative evaluation of aesthetic damage
Source: PLoS One. 2019 Dec 18;14(12):e0226322. doi: 10.1371/journal.pone.0226322 (PMC6919621; doi:10.1371/journal.pone.0226322)
Supplement: S1 Code site — 1. (ZIP) [file pone.0226322.s001.zip › [EESC_jr][Projeto]Relatorio_07_21/reports/templates_zip/gentelella-master/vendors/select2/docs/_includes/footer.html]

{% include social-buttons.html %}

Select2 is licensed under MIT, documentation under CC BY 3.0.

Maintained by Kevin Brown and Igor Vaynberg with the help of our contributors.

- Currently v4.0.3
- GitHub
- Examples
- Options
- v3.5.2 docs
- Issues
- Releases
